# Aromatic l‐amino acid decarboxylase deficiency in Taiwan

**DOI:** 10.1002/jmd2.12387

**Published:** 2023-08-02

**Authors:** Wuh‐Liang Hwu, Rai‐Hseng Hsu, Mei‐Hsin Li, Hui‐Min Lee, Hui‐An Chen, Ni‐Chung Lee, Yin‐Hsiu Chien

**Affiliations:** ^1^ Department of Pediatrics National Taiwan University Hospital Taipei Taiwan; ^2^ Department of Medical Genetics National Taiwan University Hospital Taipei Taiwan; ^3^ Graduate Institute of Integrated Medicine China Medical University Taichung City Taiwan

**Keywords:** AADC, aromatic l‐amino acid decarboxylase, Chinese, *DDC*, incidence, Taiwan

## Abstract

Aromatic l‐amino acid decarboxylase (AADC) deficiency is a rare inherited disorder that affects neurotransmitter biosynthesis. A *DDC* founder mutation c.714 + 4A > T (IVS6 + 4A > T) is prevalent in the Chinese population. This study investigated the epidemiology of AADC deficiency in Taiwan by analyzing data from National Taiwan University Hospital (NTUH), a central institution for diagnosing and treating the disease. From January 2000 to March 2023, 77 patients with AADC deficiency visited NTUH. Among them, eight were international patients seeking a second opinion, and another two had one or both non‐Chinese parents; all others were ethnically Chinese. The c.714 + 4A > T mutation accounted for 85% of all mutated alleles, and 94% of patients exhibited a severe phenotype. Of the 77 patients, 31 received gene therapy at a mean age of 3.76 years (1.62–8.49) through clinical trials, and their current ages were significantly older than those of the remaining patients. Although the combined incidence of AADC deficiency in this study (1:66491 for 2004 and later) was lower than that reported in newborn screening (1:31997 to 1:42662), case surges coincided with the launch of clinical trials and the implementation of newborn screening. Currently, many young patients are awaiting for treatment.

**Synopsis**

This study highlights the high incidence of aromatic l‐amino acid decarboxylase deficiency in Taiwan, due to the Chinese *DDC* founder mutation, and the burden associated with treating these patients in Taiwan.

## 1 INTRODUCTION

Aromatic l‐amino acid decarboxylase (AADC, EC 4.1.1.28) deficiency (MIM #608643) is a rare inherited disorder affecting neurotransmitter biosynthesis.^1^ AADC converts l‐3,4‐dihydroxyphenylalanine (levodopa) to dopamine and 5‐hydroxytryptophan to serotonin, with dopamine also serving as a precursor for epinephrine and norepinephrine biosynthesis. AADC deficiency was first identified in 1990 by Hyland and Clayton.^2^, ^3^ Patients with AADC deficiency typically present with hypotonia, hypokinesia, oculogyric crises, autonomic dysfunction in early life, and profound developmental delay.^4^, ^5^, ^6^ However, patients with milder manifestations have also been reported.^1^, ^7^, ^8^, ^9^

AADC deficiency arises from biallelic pathological mutations in the dopa decarboxylase (*DDC*) gene, which encodes the AADC enzyme.^1^ A *DDC* founder mutation c.714 + 4A > T (IVS6 + 4A > T) is prevalent in the Chinese population.^10^, ^11^ This mutation causes a 37‐nucleotide insertion from intron 6 into the *DDC* mRNA but may still allow for a small amount of normally spliced mRNA product.^12^ We initially described 20 living patients carrying this mutation, all presenting with classic symptoms, and none achieved head control, the first developmental milestone in infants.^13^ We then reviewed the natural history of 37 Chinese patients with AADC deficiency, providing further evidence of a severe phenotype associated with this splice mutation.^14^

Estimations of the proportion of the c.714 + 4A > T mutation in AADC deficiency worldwide have varied. In a 2017 review of 117 cases, 80% had severe disease manifestation, 43% were Asian, and the c.714 + 4A > T mutation was found in 36 cases (26 with homozygous mutations).^9^ In another review of 123 cases, c.714 + 4A > T, p.S250F, p.R347Q, and p.G102S together represented 57% of all mutation alleles.^1^ However, among the 63 cases published in 2020, 70% had a profound motor impairment, but the 714 + 4A > T mutation represented only 15% of all mutated alleles.^8^ Overlaps between the cases in these three articles could not be assessed.

National Taiwan University Hospital (NTUH) plays a significant role in the medical care of AADC deficiency patients in Taiwan. As medical treatment with dopamine agonists and other adjunctive drugs is ineffective for the severe form of AADC deficiency prevalent in Taiwan,^1^, ^9^ we initiated gene therapy clinical trials for the disease in 2007, with the first patient treated in 2010. The gene therapy was later proven effective by us and has been approved in Europe.^13^, ^15^, ^16^ Most patients with AADC deficiency in Taiwan and some patients in nearby countries have come to NTUH for treatment opportunities after that. In the past, diagnosing AADC deficiency depended on detecting low levels of cerebrospinal fluid homovanillic acid and 5‐hydroxyindoleacetic acid (5‐HIAA) levels, which necessitated obtaining samples through lumbar puncture.^2^, ^9^ The NTUH newborn screening center covers screening for more than one third of births in Taiwan and actively researches and develops new screening methods. After establishing that dried blood spot (DBS) 3‐o‐methyldopa (3‐OMD) can serve as a convenient biomarker for AADC deficiency,^17^ we successfully carried out a pilot newborn screening program for AADC deficiency using DBS 3‐OMD concentration from September 2013 to December 2015.^18^, ^19^, ^20^ NTUH resumed this screening program in February 2020, however, to date, it remains the only screening center in Taiwan performing this test. Due to the factors mentioned above, NTUH has amassed the majority of AADC deficiency cases in Taiwan. This article summarizes the patients with AADC deficiency who have visited NTUH. The data presented here illustrate the epidemiology of AADC deficiency and the burden associated with treating these patients in Taiwan.

## 2 MATERIALS AND METHODS

### 2.1 Study design

We reviewed internal records of patients with AADC deficiency who visited NTUH from January 2000 to March 2023. Patients' demographic data (Table 1) were verified using the hospital's electronic health record system. Collected data included birth date, sex, nationality, ethnicity, survival status, dates of last visit, *DDC* variations (NM_000790.4), and phenotype (mild or severe). If survival status or date of death was unavailable, the date of the last follow‐up was used for statistical purposes. We describe all patient outcomes as known on March 1, 2023. *DDC* variant data were also retrieved from our laboratory's internal records for verification. This study was part of the long‐term follow‐up for patients with AADC deficiency approved by the Institutional Review Board (NTUH 201303100RIND), and informed consent was obtained from all patients included in the study.

**TABLE 1:** Demographic data of the patients.

Number	Total no.	Survival	Mean age	Age range
With gene therapy	31			
		24 alive	10.1	3.0–17.7
		7 deceased	11.7	3.7–17.7
No gene therapy	38			
		18 alive	4.9	0.4–17.2
		15 deceased	3.9^1^	1.2–7.5
		5 unknown	8.3^1^	3.6–17.4
Oversea patients	8		3.7^1^	1.9–6.8
Total	77			

^1^ Calculated from the date of the last visit if the exact survival status or date of death was not known.

### 2.2 Public population data and registry

Yearly birth numbers from 1996 to 2022 were obtained from the National Development Council (https://pop-proj.ndc.gov.tw/). The Statistical Report of Rare Disease Confirmed Cases in Taiwan was downloaded from the Health Promotion Administration, Ministry of Health and Welfare (https://www.hpa.gov.tw/). The Rare Disease and Orphan Drug Act was passed in 2000, requiring doctors to register Taiwanese patients with rare diseases with the government. However, the registration system was not immediately available following the Act's passage.

### 2.3 Statistics

All numbers are expressed as mean (range), and the *t*‐test was used to compare the two groups. A *p*‐value <0.05 was considered statistically significant.

## 3 RESULTS

### 3.1 Number of patients with AADC deficiency

Between January 2000 and March 2023, 77 patients with AADC deficiency visited NTUH. The gender distribution was balanced, with 41 females (53%) and 36 males (47%). Among them, eight were international patients seeking a second opinion, and another two had one or both non‐Chinese parents; all others were ethnically Chinese. Gene therapy clinical trials for AADC deficiency began in 2010. Of the 77 patients, 31 (24 alive, 7 deceased) received gene therapy. Thirty‐eight patients never received gene therapy; 18 are alive, and 20 are deceased or lost to follow‐up. The survival status of the eight oversea patients who visited NTUH only once was unknown. The most recent (March 2023) Statistical Report of Rare Disease Confirmed Cases from the Health Promotion Administration recorded 63 patients with AADC efficiency, of whom 20 have died. Although not all patients with AADC deficiency in Taiwan visited NTUH, our case number still exceeded that of the Statistical Report of Rare Disease Confirmed Cases, likely because the latter only included Taiwanese nationals and may have missed early cases. Unfortunately, we could not obtain individual case information from the Health Promotion Administration for a detailed comparison.

### 3.2 Ages of patients with AADC deficiency

The earliest patient in this cohort was born in 1996. The mean current age of living patients who received gene therapy (*n* = 24) was 10.0 years (range: 3.0–17.7), while the mean age of deceased patients who received gene therapy (*n* = 7) was 11.7 years (range: 3.7–17.7); there was no statistical difference between the two groups (*p* = 0.39). Among those who did not receive gene therapy, the mean age of living patients (*n* = 18) was 4.9 years (range: 0.4–17.2), the mean age of death or the last visit of deceased patients (*n* = 15) was 3.9 years (range: 1.2–7.5), and mean age of the last visit of patients with unknown survival status (*n* = 13) was 5.5 years (range: 1.9–14.7). Consequently, patients who have received gene therapy survived longer than those who did not, regardless of whether they are alive, deceased, or have an unknown survival status (all *p* < 0.01).

### 3.3 Incidence of AADC deficiency in Taiwan

**FIGURE 1:**
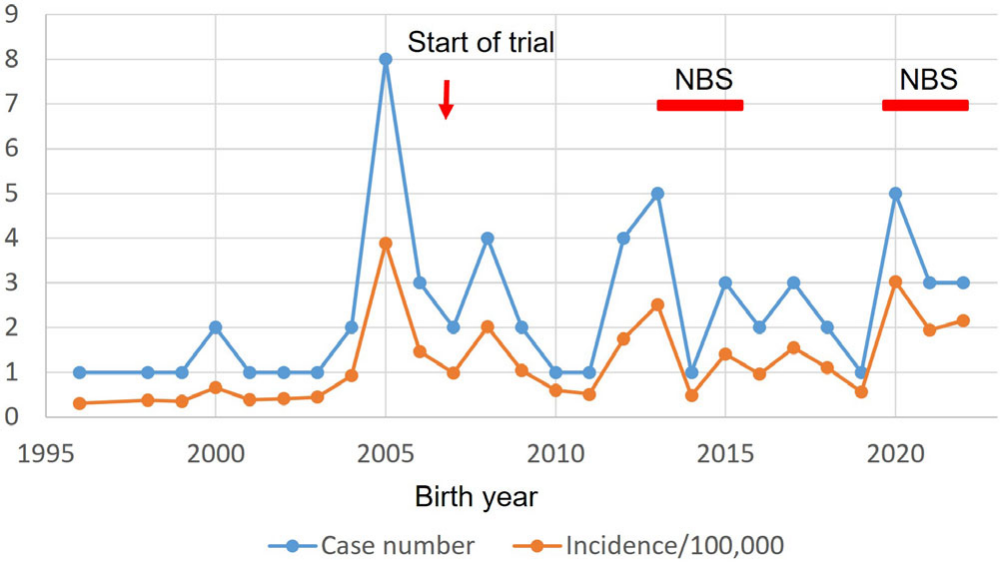
The annual occurrence of AADC deficiency in Taiwan. The number of patients (blue) and incidence per 100 000 births (orange) are plotted according to their birth year. A red arrow indicates the initiation of the gene therapy clinical trial. The period when newborn screening for AADC deficiency was conducted is denoted by red bars. AADC, aromatic l‐amino acid decarboxylase.

When we plotted the number of patients according to their year of birth from 1996 to 2002, the yearly incidence ranged from 1:25732 (3.89 per 100 000) to 1:210383 (0.48 per 100 000) (Figure 1). However, the surge in case numbers in 2005 (birth year) may be attributed to the launch of the gene therapy trial in 2007. Additionally, the surge in case numbers in 2012 and 2013 may be due to the newborn screening pilot program from September 2013 to December 2015. Finally, the peak in 2020 may be associated with the resumption of screening since February 2020. The incidence of 63 AADC deficiency cases from 1996 to 2022, with a total birth number of 5 577 906, was 1:88538. If we only considered patients born after 2004, there were 55 cases among 3 656 984 births, resulting in an incidence of 1:66491.

### 3.4 Variations in the *DDC* gene and phenotypes

Of the 77 patients, three patients discovered by newborn screening show mild but persistent elevation of 3‐OMD but are still asymptomatic, two patients had mild presentations (both showed elevation of 3‐OMD, responded to dopamine agonist, and were ambulatory at the time of diagnosis), while all other 72 patients (94%) exhibited a severe phenotype. Sequencing data were available for 59 ethnically Chinese patients, encompassing 118 alleles (*DDC* transcript number NM_000790.4). The c.714 + 4A > T mutation accounted for 85% of all alleles (Table 2). Other recurrent alleles included c.1234C > T (p.Arg412Trp; 7 alleles or 5.93%), c.1297dupA (p.Ile433AsnfsTer60; 7 alleles or 5.93%), and c.179 T > C (p.Val60Ala; 2 alleles or 1.69%). Fifteen additional variants each occurred only once in this cohort. All three asymptomatic patients identified through newborn screening had one c.714 + 4A > T mutation and another less detrimental variant (c.782‐14 T > C, c.718G > A, and c.752 T > C, respectively; Table 3). The two milder patients each had one pathogenic mutation (c.1073G > A and c.714 + 4A > T), but their other mutation was not identified (Table 3).

**TABLE 2:** List of variants and allele frequencies (percent).

Genotype	Number	Percent
c.714 + 4A > T (IVS6 + 4A > T)	85	72.03
c.1234C > T (p.Arg412Trp)	7	5.93
c.1297dupA (p.Ile433AsnfsTer60)	7	5.93
c.179 T > C (p.Val60Ala)	2	1.69
c.1058 T > C (p.Leu353Pro)	1	0.85
c.1073G > A (p.Arg358His)	1	0.85
c.170 T > C (p.Ile57Thr)	1	0.85
c.175G > A (p.Asp59Asn)	1	0.85
c.236A > G (p.Try79Cys)	1	0.85
c.296G > A (p.Gly99Asp)	1	0.85
c.304G > A (p.Gly102Ser)	1	0.85
c.339G > T (p.Glu113Asp)	1	0.85
c.436G > C (p.Gly146Arg)	1	0.85
c.58‐60delTAC (p.Y20del)	1	0.85
c.718G > A (p.Val240Ile)	1	0.85
c.752 T > C (p.Phe251Ser)	1	0.85
c.811_812delinsTG (p.Asp271Cys)	1	0.85
c.848A > C (p.Glu283Ala)	1	0.85
c.715‐14 T > C (IVS7‐14 T > C)	1	0.85
–	2	1.69
Total	118	100.00

*Note*: “–” indicates mutation not found, DDC transcript number NM_000790.4.

**TABLE 3:** Genotypes of patients with mild phenotypes.

No.	Variant 1	Variant 2 or VUS	Phenotype
1	c.714 + 4A > T	c.715‐14 T > C	NBS
2	c.714 + 4A > T	c.718G > A (p.Val240Ile)	NBS
3	c.714 + 4A > T	c.752 T > C (p.Phe251Ser)	NBS
4	c.1073G > A (p.Arg358His)	–	mild
5	c.714 + 4A > T	–	mild

*Note*: “–” indicates mutation not found, DDC transcript number NM_000790.4.

Abbreviations: NBS: newborn screening and asymptomatic; VUS, variant of unknown significance.

## 4 DISCUSSION

This single‐center study collected 77 cases of AADC deficiency, a substantial number compared to the 117,^9^ 123,^1^ and 63^8^ cases documented in three recent global registry‐ or survey‐type studies. The high incidence of the disease among the Taiwanese population and NTUH's significant role in diagnosing and treating AADC deficiency contribute to this difference. In addition, several patients were from other countries, albeit primarily ethnically Chinese and carrying the same founder mutation. The majority of the Taiwanese population originates from the Southern China population who also migrated to Southeast Asia, resulting in a high prevalence of AADC deficiency in China^21^, ^22^ and Southeastern countries.

Despite collecting a large number of AADC deficiency cases, surpassing the number from the Report of Rare Disease, the calculated incidences were still lower than those derived from newborn screening. In our pilot newborn screening study for AADC deficiency, four cases were detected among 127 987 newborns, with an incidence of 1:31997.^17^ If we exclude the one asymptomatic case among these four cases, the incidence would be 1:42662. In the current study, focusing on 2004 onward, the combined incidence of all cases was 1:66491, suggesting that we likely missed 35%–50% of AADC deficiency cases in Taiwan. There should be few diagnosed cases in Taiwan who were unaware of the gene therapy clinical trial or did not want to contact NTUH. This implies that some patients may have never been diagnosed. This hypothesis is further supported by the surge in case numbers coinciding with the gene therapy clinical trial launch and the implementation of newborn screening.

Although relatively rare compared to the classic phenotype, patients with milder diseases were still present in this cohort. Two patients in our cohort could walk at the time of diagnosis. Intriguingly, whole exome sequences revealed that both individuals had one mutation not found, could be resulting from a splice mutation that reduced the DDC protein product. The three asymptomatic cases detected by newborn screening all had a mutation predicted to be mild.^20^ Since we did not identify these mild mutations in other patients, predicting their age of symptom onset or the likelihood of developing symptoms remains uncertain.

**FIGURE 2:**
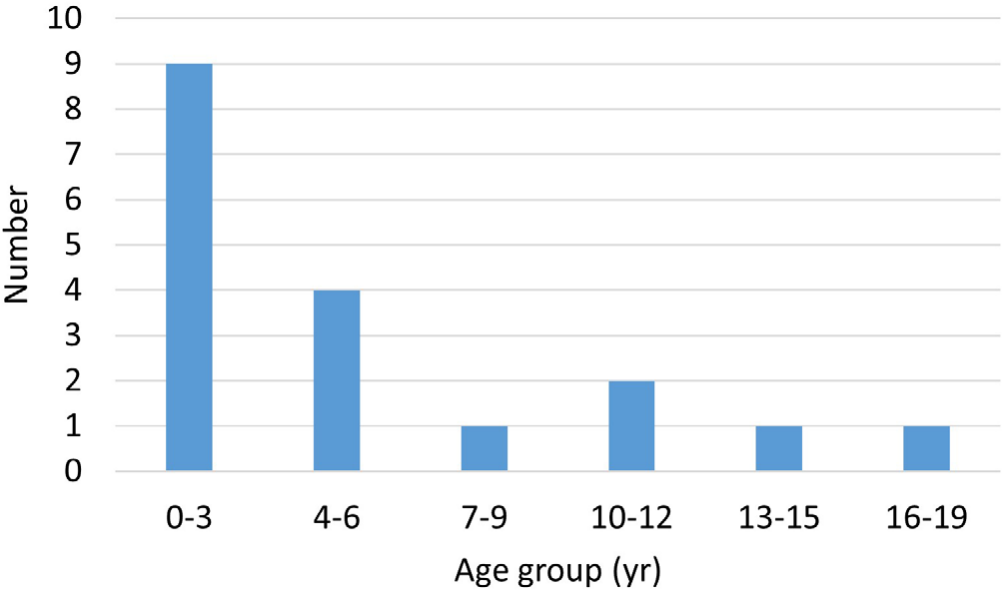
Age distribution of 18 living patients with AADC deficiency who have not received gene therapy. AADC, aromatic l‐amino acid decarboxylase.

Living patients who have not yet been treated with gene therapy are either awaiting treatment or are ineligible due to parental preferences, old age, or unsuitable general conditions. However, we noticed that half of the untreated patients were aged 0–3 years, and 70% were aged 0–6 years (Figure 2), indicating that most were young. Although the age at diagnosis was not included in this study due to frequent data gaps, many young patients who have been diagnosed early are now awaiting treatment. Therefore, we hope gene therapy will be approved in Taiwan in the near future. However, considering the high cost of gene therapy products,^23^ the burden of treating AADC deficiency in Taiwan is significant.

## 5 CONCLUSION

This study highlights the high incidence of AADC deficiency in Taiwan and neighboring countries due to the Chinese *DDC* founder mutation, with gene therapy significantly altering the natural course of affected patients. The findings also indicate a considerable number of undiagnosed patients, emphasizing the importance of enhancing disease awareness and implementing newborn screening to improve diagnostic rates.

## CONFLICT OF INTEREST STATEMENT

Wuh‐Liang Hwu participated as an advisory board member, received consulting fees, and was a speaker for PTC Therapeutics. In addition, he was a grant recipient for PTC Therapeutics and a research investigator for PTC Therapeutics. Rai‐Hseng Hsu, Mei‐Hsin Li, Hui‐Min Lee, Hui‐An Chen, Ni‐Chung Lee, and Yin‐Hsiu Chien declare no conflict of interest.

## ETHICS STATEMENT

This study was part of the long‐term follow‐up for patients with AADC deficiency approved by the Institutional Review Board (NTUH 201303100RIND), and informed consent was obtained from all patients included in the study.

## Data Availability

The population data used in this study were obtained from the National Development Council (https://pop-proj.ndc.gov.tw/; Yearly birth numbers) and the Health Promotion Administration (https://www.hpa.gov.tw/; Report of Rare Disease).
